# Combination therapy improves survival prognosis in anti-MDA5-antibody-positive dermatomyositis patients: a single-center retrospective study

**DOI:** 10.3389/fimmu.2025.1658547

**Published:** 2025-11-17

**Authors:** Congcong Gao, Gaohui Wei, Chaoying Li, Chunyi Zhang, Chenqiong Wang, Ruxu Li, Kebing Shen, Zhaohui Zheng

**Affiliations:** 1 Department of Rheumatology, The First Affiliated Hospital of Zhengzhou University, Zhengzhou, China; 2 Department of Clinical Laboratory, The First Affiliated Hospital of Zhengzhou University, Zhengzhou, China

**Keywords:** anti-melanoma differentiation-related gene 5, dermatomyositis, combination therapy, mortality, risk factor, co-infection

## Abstract

**Background:**

Anti-melanoma differentiation-associated gene 5 antibody-positive dermatomyositis (MDA5+DM) is associated with poor prognosis and high mortality, presenting significant challenges for treating this intractable disease. This study aimed to compare the efficacy and safety of calcineurin inhibitor monotherapy (CNI) versus combination therapy [CNI and tofacitinib (TOF) or cyclophosphamide (CTX)] as initial immunosuppressive regimens for MDA5+DM.

**Methods:**

In this retrospective observational study, MDA5+DM patients from the First Affiliated Hospital of Zhengzhou University between August 2019 and June 2024 were included. Patients were categorized into three groups according to the different immunosuppressive regimens. One-year mortality and the potential risk factors for death was analyzed using the Kaplan-Meier survival analysis and Cox proportional hazards regression, respectively.

**Results:**

A total of 152 patients were divided into CNI group(n=49, 32.2%), CNI+TOF group(n=52, 34.2%), and CNI+CTX group(n=51, 33.6%). The 1-year survival rate was significantly lower in the CNI group compared to in the combination therapy groups (logrank P = 0.032). However, the CNI+CTX group showed a higher overall infection rate compared to CNI and CNI+TOF group (51.0% vs 28.6% vs 32.7%, p=0.048). Multivariate analysis identified combination therapy and higher CD8+ T cells act as protective factors, whereas co-infection is a major predictor of mortality.

**Conclusions:**

In our study, combination therapy may improve survival prognosis in MDA5+ DM patients. Nevertheless, vigilant monitoring for opportunistic infections during treatment is essential.

## Introduction

Dermatomyositis (DM) is a systemic autoimmune inflammatory disease characterized by proximal muscle weakness and cutaneous manifestations (1). Anti-melanoma differentiation-associated gene 5-positive dermatomyositis (MDA5+DM) is a distinct subtype of DM, frequently associated with rapidly progressive interstitial lung disease (RPILD) and high mortality (2). Emerging evidence indicates that the 6-month mortality rate for MDA5+DM patients with RPILD ranges from 50% to 70% (3–5).

The treatment strategies for MDA5+DM patients are determined based on disease severity, prognostic risk factors, and indicators of disease progression. The initial treatment for MDA5+DM commonly involves glucocorticoids combined with immunosuppressive agents, particularly calcineurin inhibitors, but its therapeutic efficacy remains rather limited (6). Currently, the recommended induction treatment protocol includes a combination therapy of high-dose systemic glucocorticoids and immunosuppressants (e.g., calcineurin inhibitors and cyclophosphamide). Administering this regimen at an early stage has shown the greatest potential to improve survival among patients with RPILD (7–9). However, a recent multicenter longitudinal cohort study of 115 MDA5+DM patients reported contradictory findings. The investigation failed to show improved survival with a triple-combination therapy consisting of high-dose corticosteroids, tacrolimus, and intravenous cyclophosphamide (10). Additionally, the triple-combination therapy increases infection risks (especially viral and fungal), with co-infections further exacerbating mortality (7, 11). Therefore, the initial triple-combination therapy may not be appropriate for all patients, necessitating careful evaluation of benefits and risks before initiation.

Despite aggressive immunosuppressive regimens, a certain group of patients still experience unremitting disease progression and premature death, highlighting the pressing need for alternative or supplementary therapeutic approaches. In recent studies, hyperactivation of the type I IFN signaling pathway has emerged as a key factor closely associated with the pathogenesis of MDA5+DM (2, 12). Tofacitinib, a potent inhibitor of the JAK-STAT signaling and interferon pathway, shows a promising treatment option for MDA5+DM-associated interstitial lung disease (ILD). A previous single-center trial showed that tofacitinib improved survival in MDA5+DM-ILD compared to historical controls (100% vs 78%) (13). These compelling results have firmly established the groundwork for the clinical application of tofacitinib, further supported by a large multicenter cohort showing greater benefits for 1-year lung transplantation-free survival versus calcineurin inhibitors (14). Another retrospective cohort of MDA5+DM patients demonstrated superior efficacy of combined calcineurin and tofacitinib therapy versus calcineurin inhibitor monotherapy (12).

At present, large-scale studies directly comparing the efficacy and safety of calcineurin inhibitor monotherapy, calcineurin inhibitor combined with tofacitinib or cyclophosphamide in the treatment of MDA5+DM are notably scarce. This research is designed to evaluate and contrast the 1-year mortality and infection risks among these regimens to inform more effective treatment strategies.

## Methods

### Patients

In this retrospective study, adult MDA5+ DM patients admitted to the First Affiliated Hospital of Zhengzhou University between August 2019 and June 2024 were included. A diagnosis of MDA5+DM was based on the 2017 EULAR/ACR IIM classification criteria or the 2018 EMNC DM criteria (15, 16). Patients were excluded if they met any of the following criteria: (1) undergoing other forms of drug treatments other than the three predefined regimens; (2) having received immunosuppressive therapy for less than one month; (3) presenting with severe coexisting infection at baseline; (4) having incomplete case data; (5) being lost to follow-up; (6) having a concurrent diagnosis of other autoimmune diseases. This study was approved by the Ethics Committee of the First Affiliated Hospital of Zhengzhou University (2020-KY-522).

### Clinical and laboratory data

Baseline characteristics including demographics, clinical features, comorbidities, laboratory data and treatments were acquired from the patients’ electronic medical records, and we have no direct interaction with these patients. The laboratory data was routine clinical measurements, ordered solely by treating physicians based on clinical needs. All testing including serum KL-6 levels and lymphocyte subset was performed by the hospital’s Central Clinical Laboratory staff, not by the study authors. We conducted only retrospective analysis on anonymized data extracted from the Laboratory Information System. Anti-MDA5 antibody was determined using ELISA kits (MBL, Japan). The presence of ILD was evaluated via chest high-resolution computed tomography(HRCT). Rapidly progressive ILD (RPILD) was defined as acute progressive dyspnea and hypoxemia within 4 weeks from the onset of respiratory symptoms, accompanied by aggravation of ILD on HRCT (17). Patient treatment parameters included the daily dosage of glucocorticoids (GCs), immunosuppressants (IS) including cyclophosphamide (CTX), calcineurin inhibitors (CNI), and tofacitinib. All of the patients received either tacrolimus or cyclosporine as a calcineurin inhibitor. Patients were followed up for at least one year.

### Statistical analysis

Statistical analysis was performed with IBM SPSS software (version 26.0, Armonk, NY, USA) and GraphPad Prism software version 9.0 (GraphPad Software, San Diego, California, USA). Continuous variables with non-normally distributions were presented as medians and interquartile ranges (IQR), with Mann-Whitney U test used to assess the difference between groups. While categorical variables were summarized as numbers and percentages, and subsequently compared using the Chi-square test and Fisher’s exact test. Cumulative survival rates were compared using Kaplan-Meier analysis, and the log-rank test was used to test for significant differences between groups. The variables showing statistical significance (P<0.05) in the univariate analysis were included in the Cox multivariate regression analysis to identify independent risk factors for death in MDA5+DM patients. In the Cox regression analysis, the proportional hazards assumption was tested using the Schoenfeld residual method. For all analyses, two-sided P-values < 0.05 were considered statistically significant.

## Results

### Baseline data description

**Figure 1:**
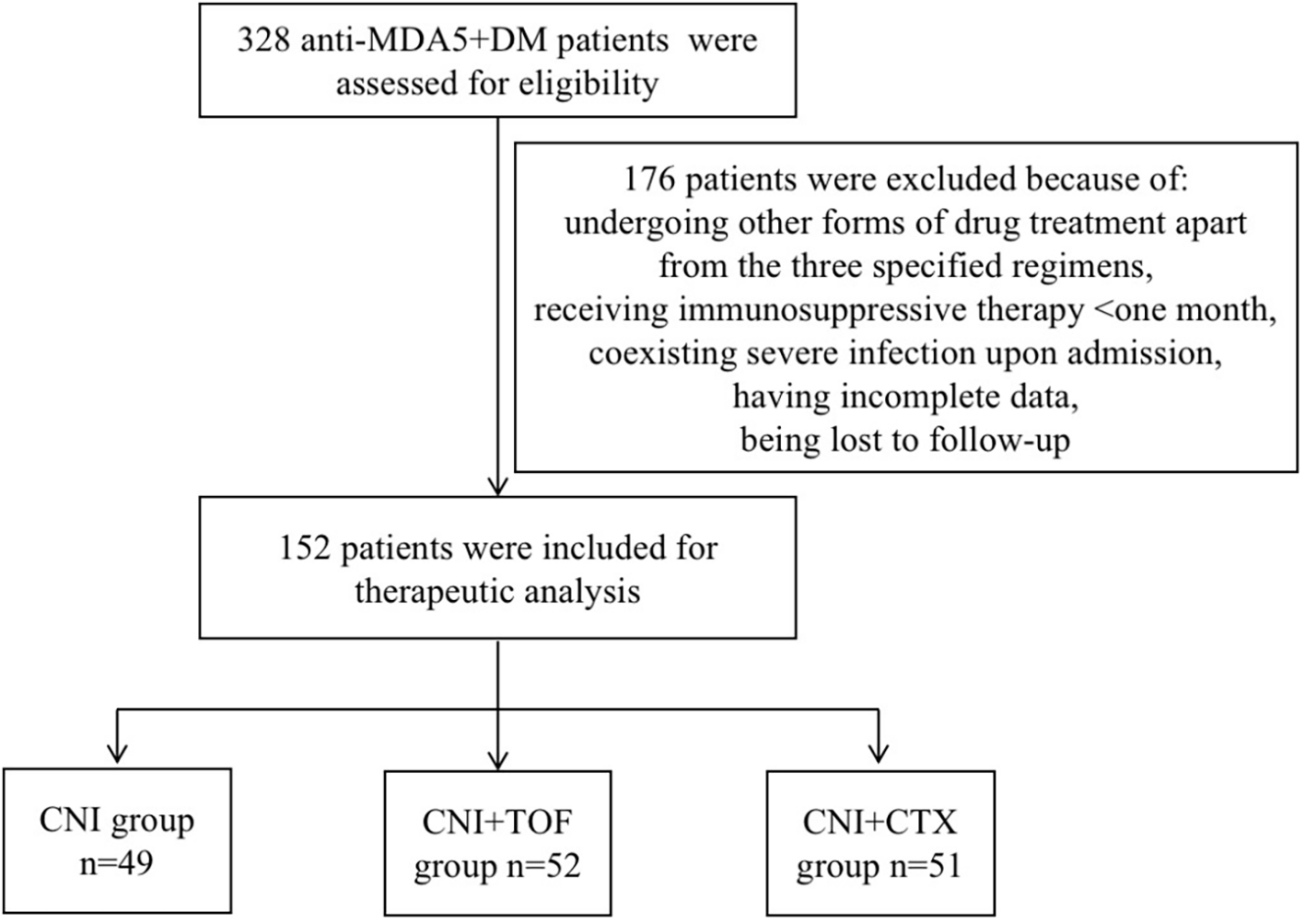
Flow chart of analyzed patients. MDA5+DM, anti-melanoma differentiation-associated gene 5 antibody-positive dermatomyositis; CNI, calcineurin inhibitors; CTX, cyclophosphamide; TOF, tofacitinib.

From August 2019 to June 2024, we reviewed 328 cases with MDA5+ DM from the departments of rheumatology, respiratory and ICU at the First Affiliated Hospital of Zhengzhou University. In total, 176 patients were excluded from the study. Their exclusion was due to undergoing other forms of drug treatment apart from the three specified regimens, receiving immunosuppressive therapy for less than one month, coexisting severe infection upon admission, having incomplete data, or being lost to follow-up. Finally, 152 patients were included in the analysis. Every patient among them suffers from interstitial lung disease. According to initial immunosuppressive regimens after admission, these patients were divided into CNI group (n=49, 32.2%), CNI+TOF group(n=52, 34.2%), and CNI+CTX group(n=51, 33.6%). The screening process of the patients were shown in **Figure 1**.

The baseline demographic features, clinical manifestations, laboratory data and treatments between three groups were recorded in **Tables 1** and **2**. The three groups had similar ages, gender distributions and disease course. Dyspnea (p=0.011) and myasthenia (p=0.035) were more prevalent in the CNI+CTX group, while RPILD incidence did not differ (p=0.160). All patients received GCs, with comparable rates of maximum methylprednisolone (MP) dosage ≥80 mg/day at baseline (p=0.249). Laboratory results demonstrated that the CNI+CTX group had significantly lower lymphocyte counts (p=0.045) and albumin (ALB) levels (p=0.021), as well as significantly higher aspartate aminotransferase (AST) levels (p=0.024).

**Table 1:** Clinical characteristics of MDA5+DM patients at baseline.

Variables	CNI group(n=49)	CNI+TOF group(n=52)	CNI+CTX group(n=51)	P value
Age, years, median(IQR)	54.0(40.5-59.0)	52.0(44.5-58.0)	51.0(41.0-58.0)	0.846
Female, n%	32(65.3)	38(73.1)	29(56.9)	0.227
Disease course, months, Median (IQR)	2(1-4)	2(1-4)	2(1-4)	0.872
Fever, n%	24(49.0)	26(50.0)	22(43.1)	0.757
Arthritis, n%	23(46.9)	25(48.1)	19(37.3)	0.483
Dyspnea, n%	9(18.4)	6(11.5)	18(35.3)	0.011*
RPILD, n%	12(24.5)	11(21.2)	19(37.3)	0.160
Gottron papule, n%	22(44.9)	22(42.3)	17(33.3)	0.464
V sign, n%	15(30.6)	13(25.0)	10(19.6)	0.449
Shawl sign, n%	10(20.4)	11(21.2)	9(17.6)	0.896
Superficial erosion and ulcer, n%	10(20.4)	5(9.6)	12(23.5)	0.155
Mechanic’s hands, n%	13(26.5)	16(30.8)	12(23.5)	0.709
Heliotrope rash, n%	24(49.0)	23(44.2)	21(41.2)	0.734
Myasthenia, n%	11(22.4)	14(26.9)	23(45.1)	0.035*
Myalgia, n%	13(26.5)	11(21.2)	11(21.6)	0.779
Maximum MP dosage≥ 80mg/day, n%	22(44.9)	19(36.5)	27(52.9)	0.249

*Values statistically significant at p<0.05.

MDA5+DM, anti-melanoma differentiation- associated gene 5 antibody-positive dermatomyositis; RPILD, rapidly progressive interstitial lung disease; MP, methylprednisolone; CNI, calcineurin inhibitors; CTX, cyclophosphamide; TOF, tofacitinib.

**Table 2:** Laboratory characteristics of anti-MDA5+DM patients at baseline.

Variables	CNI group(n=49)	CNI+TOF group(n=52)	CNI+CTX group(n=51)	P value
Neutrophile granulocyte count, × 10^9^/L median (IQR)	3.5(2.6-5.3)	3.6(2.9-6.0)	3.7(2.5-4.7)	0.470
Lymphocyte count,× 10^9^/L, median (IQR)	0.9(0.6-1.2)	0.9(0.7-1.2)	0.7(0.5-1.0)	0.045*
NLR, median (IQR)	4.0(2.7-6.9)	3.7(2.5-6.8)	4.6(2.7-7.1)	0.537
LDH,U/L, median (IQR)	353.0(251.5-440.0)	325.0(282.3-419.8)	341.0(283.0-414.0)	0.974
CK,U/L, median (IQR)	97.0(41.0-189.5)	49.0(31.5-117.5)	82.0(50.0-129.0)	0.058
ALT,U/L, median (IQR)	33.0(18.5-71.5)	41.5(26.8-74.8)	56.0(24.0-92.0)	0.210
AST,U/L, median (IQR)	45.0(24.5-88.5)	41.0(33.0-64.3)	68.0(38.0-119.0)	0.024*
ALB, g/L, median (IQR)	35.3(30.4-39.5)	36.1(32.7-38.5)	33.1(30.0-36.5)	0.021*
FET, ng/mL, median (IQR)	580.0(308.6-11538.0)	656.3(331.1-975.5)	934.4(429.0-1385.6)	0.229
KL-6,U/mL, median (IQR)	664.0(473.0-1046.0)	835.5(584.5-1244.3)	818.0(625.0-1300.0)	0.086
Anti-MDA5 antibody titer, U/mL, median (IQR)	181.1(142.4-204.7)	166.8(123.3-195.6)	178.9(162.3-203.9)	0.124
ESR, mm/h, median (IQR)	26.0(12.5-41.0)	27.5(16.0-45.8)	34.0(21.0-50.0)	0.124
CRP, mg/L, median (IQR)	1.7(0.8-10.7)	4.7(0.8-10.8)	3.4(0.8-11.6)	0.564
IgG, g/L, median (IQR)	13.0(10.8-15.9)	12.2(10.1-15.5)	13.8(11.0-15.3)	0.503
IgA, g/L, median (IQR)	2.5(2.0-3.4)	2.6(2.0-3.1)	3.1(2.1-3.7)	0.146
IgM, g/L, median (IQR)	1.2(0.8-1.6)	1.3(0.9-2.1)	1.3(0.9-1.8)	0.514
CD3+T cells,/uL, median (IQR)	501.0(383.3-829.5)	599.5(419.5-858.0)	485.0(316.0-721.0)	0.120
CD4+T cells,/uL, median (IQR)	320.0(210.0-468.0)	359.0(256.5-474.0)	325.0(185.0-413.0)	0.383
CD8+T cells,/uL, median (IQR)	166.0(93.9-276.5)	179.5(130.0-309.0)	152.0(99.0-258.0)	0.240
CD19+B cells,/uL, median (IQR)	108.0(65.5-236.5)	138.5(94.3-249.3)	112.9(69.0-185.0)	0.307

*Values statistically significant at p<0.05.

NLR, neutrophil/lymphocyte ratio; LDH, lactate dehydrogenase; CK, creatine kinase; ALT, Alanine aminotransferase; AST, aspartate aminotransferase; ALB, albumin; FET, ferritin; KL-6, Krebs von den Lungen-6; ESR, erythrocyte sedimentation rate; CRP, C-reactive protein; IgG, Immunoglobulin G; IgA, Immunoglobulin A; IgM, Immunoglobulin M;CNI, calcineurin inhibitors; CTX, cyclophosphamide; TOF, tofacitinib.

### Serious infection events

Serious infection events within 1 year were summarized in **Table 3**. In total, 14(28.6%) patients in the CNI group, 17(32.7%) patients in the CNI+TOF group and 26 (51.0%) patients in the CNI+CTX group had infectious SAEs (P = 0.048). Cytomegalovirus (CMV) was the most common viral infection, with Aspergillus and Pneumocystis jirovecii (PJP) as major fungal pathogens. The CNI+CTX group had higher rates of viral, bacterial, and fungal infections, though not statistically significant.

**Table 3:** Serious infection events within 1 year.

Variables	CNI group(n=49)	CNI+TOF group(n=52)	CNI+CTX group(n=51)	P value
Overall infection, no. of patients (%)	14(28.6)	17(32.7)	26(51.0)	0.048*
Pathogens, no. of events (%)				
Virus	6(12.2)	12(23.1)	15(29.4)	0.111
Bacteria	8(16.3)	5(9.6)	13(25.5)	0.101
Fungi	8(16.3)	9(17.3)	16(31.4)	0.122

*Values statistically significant at p<0.05.

CNI, calcineurin inhibitors; CTX, cyclophosphamide; TOF, tofacitinib.

### Risk factors for mortality

**Figure 2:**
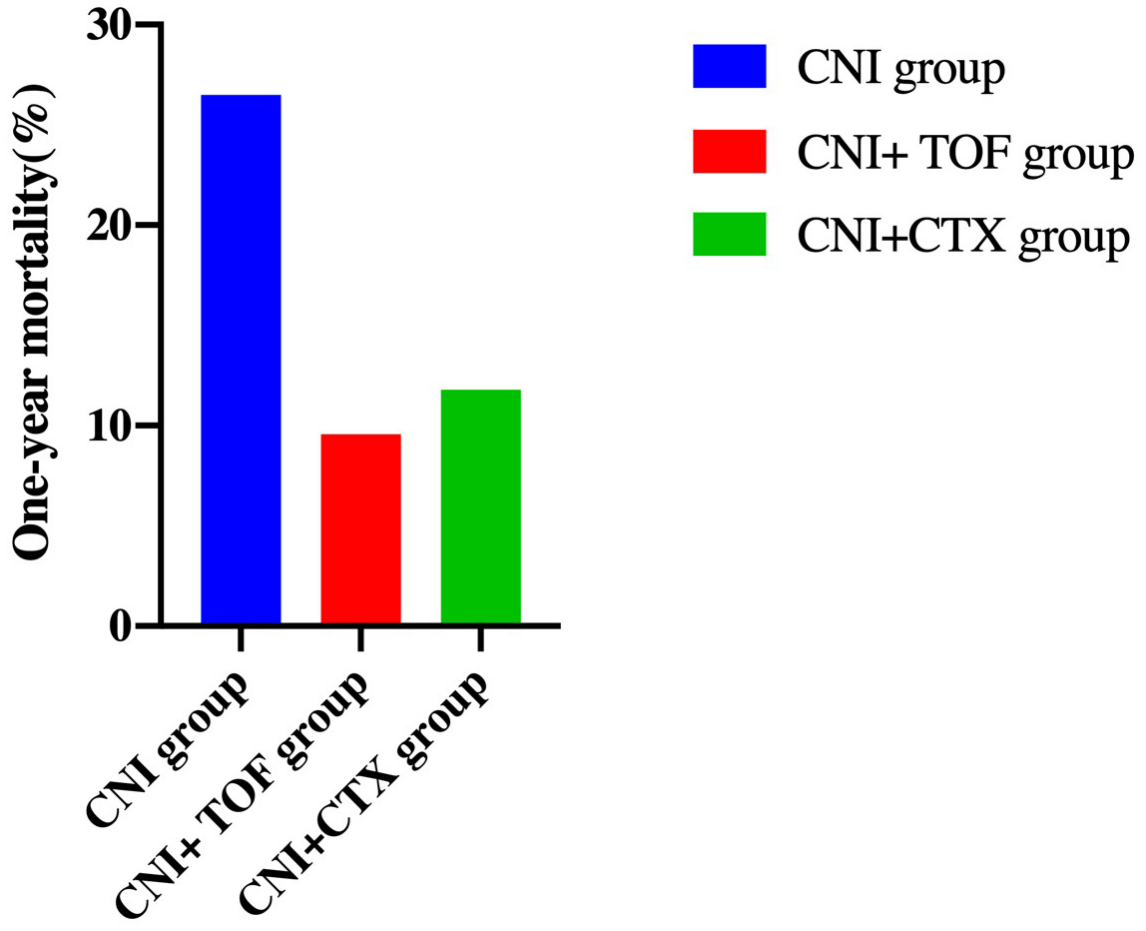
One-year mortality in each group of patients. Patients treated with CNI showed a higher 1-year mortality than those treated with CNI+TOF and CNI+CTX (26.5% vs 9.6% vs 11.8%, p=0.042). CNI, calcineurin inhibitors; CTX, cyclophosphamide; TOF, tofacitinib.

**Figure 3:**
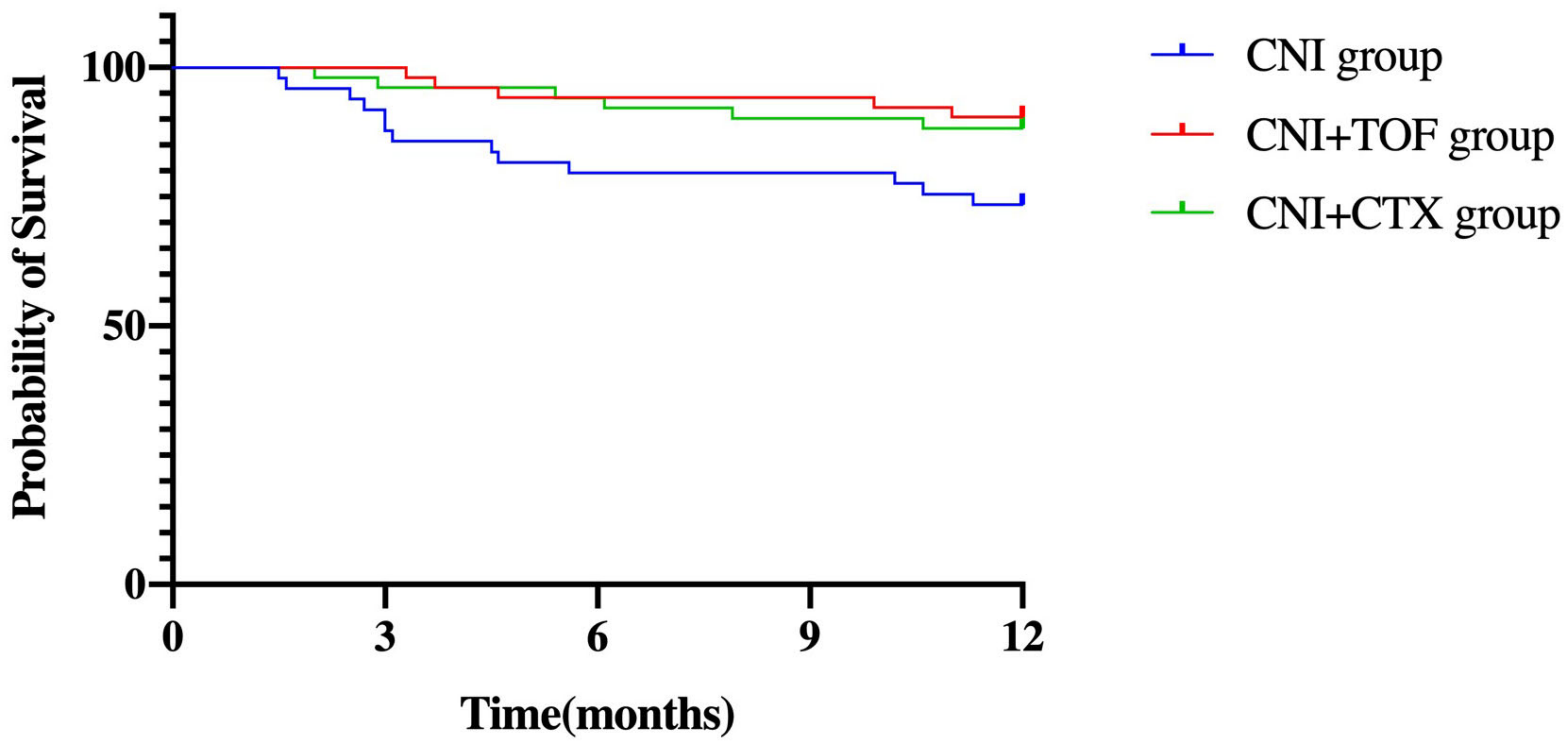
Survival in each group of patients within one year. log-rank *p* = 0.032. Patients treated with CNI+TOF or CNI+CTX had significantly better 1-year survival than those treated with CNI (P = 0.032 by the log-rank test). CNI, calcineurin inhibitors; CTX, cyclophosphamide; TOF, tofacitinib.

Cumulatively, a total of 24(15.8%) patients died within one year. The CNI group showed a higher 1-year mortality compared to CNI+TOF and CNI+CTX group (26.5% vs 9.6% vs 11.8%, p=0.042) (**Figure 2**). While, the 1-year survival rate was significantly lower in the CNI group compared to combination groups (logrank P = 0.032) (**Figure 3**).

We assessed the differences between patients survived or died within one year, and further, conducted the potential risk factors for death. In the univariable analysis, age, disease course, dyspnea, RPILD, AST, ESR, CRP, CD4+T cells, CD8+T cells, co-infection and treatments showed significant association with death(all p<0.05). Multivariate analysis showed CNI+TOF(HR = 0.309, 95% CI: 0.100-0.956, p=0.041, compared to CNI), CNI+CTX(HR = 0.150, 95% CI: 0.046-0.491, p=0.002, compared to CNI), and CD8+T cells (HR = 0.993, 95% CI: 0.987-1.000, p=0.040) as protective factors, while, co-infection (HR = 2.917, 95% CI: 1.073-7.933, p=0.036) was a risk factor (**Table 4**).

**Table 4:** Risk factors for all-cause death within 1 year determined by multivariable Cox proportional-hazards model.

Covariates	Hazard ratio (95% CI)	P value
CNI+TOF vs. CNI	0.309 (0.100-0.956)	0.041*
CNI+CTX vs. CNI	0.150 (0.046-0.491)	0.002*
Age	1.009 (0.960-1.061)	0.727
Disease course	0.800 (0.586-1.090)	0.158
Dyspnea	2.047 (0.669-6.264)	0.209
RPILD	1.945 (0.646-5.860)	0.237
AST	1.000 (0.998-1.002)	0.806
ESR	1.008 (0.982-1.035)	0.543
CRP	1.000 (0.981-1.020)	0.983
CD4+T cells	1.001 (0.998-1.003)	0.620
CD8+T cells	0.993 (0.987-1.000)	0.040*
co-infection	2.917 (1.073-7.933)	0.036*

*Values statistically significant at p<0.05.

CNI, calcineurin inhibitors; CTX, cyclophosphamide; TOF, tofacitinib; RPILD, rapidly progressive interstitial lung disease; AST, aspartate aminotransferase; ALB, albumin; ESR, erythrocyte sedimentation rate; CRP, C-reactive protein.

## Discussion

In this single-center retrospective study on MDA5+DM-ILD patients, combination therapy (calcineurin inhibitor and tofacitinib or cyclophosphamide) as initial immunosuppressive regimen was found to significantly reduce the 1-year mortality risk compared to calcineurin inhibitor monotherapy. However, the incidence of overall infection was the highest in the CNI+CTX group than the other groups. CNI monotherapy and co-infection were major mortality predictors, while CD8+T cells were protective.

The etiology of MDA5+DM remains unclear. Although environmental-genetic interactions are implicated, the underlying mechanisms remain incompletely understood, involving complex immune cell interactions (T cells, B cells, neutrophils, macrophages, natural killer cells). Notably, accumulating evidence has demonstrated that overactivation of the type I interferon (IFN) signaling pathway is strongly associated with the pathogenesis of MDA5+DM (12). Guided by the current understanding of its pathogenesis, the existing treatment for MDA5+DM-ILD typically involves a combination of high-dose glucocorticoids, calcineurin inhibitors, and intravenous cyclophosphamide (7). In parallel, researchers have explored alternative therapeutic modalities, notably the triple combination of glucocorticoids, calcineurin inhibitors, and tofacitinib (12). These clinical investigations have demonstrated that combined immunotherapeutic regimens confer a distinct advantage, markedly improving survival outcomes of MDA5+DM patients. Consistent with the previous studies, our results showed that the triple combination therapy can remarkably enhance the survival rates of these individuals.

However, for such MDA5+DM patients, intensive immunosuppressive regimens may increase the risk of opportunistic infections. In our research, the overall infection incidence among these patients was strikingly high, reaching 37.5%. Notably, the infection rate in the CNI + CTX group was even more pronounced, surging to 51%. Among these infectious cases, specific pathogens emerged as the most common culprits, prominently including Aspergillus, PJP, and CMV, which is consistent with previous studies (7, 18, 19). Specifically, for patients undergoing combined immunosuppressive regimens, the CMV infection rate reached a staggering 85% (7). Analogously, the incidence of PJP among MDA5+DM is equally concerning, soaring to 48.4% (20). Moreover, these infections often served as a catalyst, precipitating the exacerbation of ILD activity, thereby increasing the risk of mortality.

As expected, infection proved to be a pivotal risk factor for mortality among MDA5+DM patients in our study. This finding aligns closely with the results reported in prior studies (19, 20). Notably, a staggering 62.5% of the patients who succumbed to the disease had concurrent infections. Prophylactic medications and careful monitoring for opportunistic infections should be considered as a routine in the management of MDA5+DM. Previous evidence has demonstrated that prophylactic trimethoprim- sulfamethoxazole(TMP-SMZ) treatment was associated with a reduced incidence of PJP and a lower mortality rate among MDA5+DM patients (21). This intervention holds significant promise in enhancing patient outcomes and improving long-term prognosis for this vulnerable population.

It is noteworthy that peripheral lymphopenia is a unique laboratory feature of MDA5+DM. This condition is characterized by simultaneous decreases in both CD4+ and CD8+ T cells, with the reduction being especially pronounced in patients with RPILD, and indicates that T cells play an important role in the development of MDA5+DM. Additionally, lymphopenia has emerged as a significant risk factor for patient mortality. Previous research has postulated that lymphocytes migrate to the lungs to engage in the local immune response, thereby leading to a reduction in peripheral blood lymphocytes (22, 23). This redistribution of lymphocytes may represent an adaptive immune mechanism, where immune cells are mobilized to the site of inflammation, yet it simultaneously results in lymphocytopenia in the circulatory system. The roles of CD4+T lymphocytes in MDA5+DM have been widely studied and are well recognized. Emerging evidence also suggests that CD8+ T cells play a critical role in MDA5+DM (24). In our study, we also found that CD4+T cells and CD8+T cells in the death group were lower via the univariable analysis, and further multivariable analysis revealed that more CD8+T cells might improve survival prognosis in MDA5+DM patients. It is a well-established fact that CD8+T cells, also known as cytotoxic T cells, possess the remarkable ability to directly identify and eliminate virus-infected cells. Through the release of potent cytotoxic agents, including perforin and granzymes, these cells initiate a cascade of events that culminate in the apoptosis of target cells, effectively purging the body of invading pathogens. CD8+T cells from patients with MDA5+DM showed significant exhausted phenotype, and increased exhausted CD8+T cells were associated with high risk of pulmonary fungal infection (25). An increased number of circulating CD8+T lymphocytes acts as a formidable bulwark, significantly bolstering the overall defense mechanisms against various infectious agents. This enhanced immunological surveillance and response capacity translate into a substantial decrease in mortality rates associated with infectious diseases. Evidently, CD8+ T cells play a central and indispensable role in ensuring survival in the face of infectious threats. The mechanism for CD8+T cells in MDA5+DM is complex and warrants further investigation.

Our study has several limitations. Firstly, it was a retrospective study conducted in one hospital, and the sample size of the cohort was relatively small, which might have restricted the detection of certain potential positive outcomes. In the future, randomized controlled trials with larger samples are needed to explore the therapeutic value of triple therapy in MDA5+DM. Secondly, the pulmonary function, which serve as crucial indicator of the severity of lung lesions, was not routinely recorded in this real-world cohort. Thirdly, adverse events might not be completely recorded. Lastly, the study only involved a Chinese population, potentially limiting its generalizability. Further prospective studies with larger cohorts are warranted to confirm our findings.

## Conclusions

In this retrospective cohort study, we discovered that combination therapy may enhance the survival prognosis of patients with MDA5+ DM. Nevertheless, vigilant monitoring for opportunistic infections during treatment is essential. This comprehensive approach ensures both the therapeutic efficacy and the overall safety of patients, striking a crucial balance between treatment benefits and potential complications.

## Data Availability

The raw data supporting the conclusions of this article will be made available by the authors, without undue reservation.
